# From pantry to physiology: A narrative review linking food insecurity to biological dysfunction

**DOI:** 10.1016/j.dialog.2026.100308

**Published:** 2026-05-12

**Authors:** Liahm Blank, Joshua Khorsandi, Nicole Kang, Kaloyan Momchilov, Itai Blank, Kavita Batra

**Affiliations:** a Kirk Kerkorian School of Medicine at UNLV, 625 Shadow Ln, Las Vegas, NV 89106, USA; b University of Nevada, Las Vegas 4505 S Maryland Pkwy, Las Vegas, NV 89154, USA; c Department of Medical Education and Office of Research, Kirk Kerkorian School of Medicine at UNLV, 1701 W. Charleston Blvd., Las Vegas, NV 89106, USA

**Keywords:** Nutrition, Metabolism, Morbidity, Mortality, Physiology, Policy, Equity

## Abstract

**Background:**

Food insecurity is an escalating public health and social equity challenge with measurable biological consequences. Defined as limited or uncertain access to nutritionally adequate and safe foods, it affects millions of households in the United States and is increasingly recognized as a driver of chronic disease. While research on food insecurity has expanded across public health, nutrition, and social science, understanding of its biological mechanisms remains fragmented.

**Methods:**

Using the Scale for the Assessment of Narrative Review Articles (SANRA) guidelines, this narrative review synthesizes interdisciplinary evidence connecting food insecurity to physiological dysfunction, integrating findings from epidemiologic, clinical, and mechanistic studies. It explores the following: (1) general health and life expectancy; (2) cardiovascular disease; (3) diabetes; (4) nutrition, weight, and metabolic health; (5) mental health and sleep; and (6) immune function. Across these domains, chronic nutritional instability and psychosocial stress are linked to hormonal and inflammatory dysregulation, metabolic disturbance, and increased allostatic load.

**Results:**

These mechanisms demonstrate that food insecurity operates not only as a social condition but as a biologically embedded stressor. Further coordination is needed to address both biological and structural drivers of disease.

**Conclusions:**

Food insecurity should be recognized as a modifiable determinant of physiological health and health equity. These findings align with global health priorities, including SDG 2 (Zero Hunger), SDG 3 (Good Health and Well-Being), and SDG 10 (Reduced Inequalities), highlighting food insecurity as a key driver of health disparities. Integrating insights from nutrition science, medicine, and social policy is essential to develop effective, multisector interventions. This review underscores the need for cross-sector collaboration to bridge nutrition science, medicine, and social policy, reframing food insecurity as a modifiable determinant of physiological health and health equity.

## Highlights

• Food insecurity is a biologically embedded stressor, not solely a socioeconomic condition.
• Food insecurity increases allostatic load, inflammation, and neuromuscular dysregulation.
• Food insecurity contributes to cardiometabolic, mental health, and immune dysfunction.
• Understanding causal pathways can guide policies and interventions addressing food insecurity.

## 1 Introduction

Food insecurity, defined by the U.S. Department of Agriculture (USDA) as the limited or uncertain availability of nutritionally adequate and safe foods, represents one of the most pervasive social determinants of health in the United States [1]. Despite being a high-income nation, the United States continues to experience significant disparities in food access and nutritional quality. In 2023, approximately 47.4 million Americans, including 7.2 million children, lived in food-insecure households, marking one of the highest rates since the early 2010s [1]. The drivers of food insecurity are multifactorial, rooted in poverty, high food costs, housing instability, transportation barriers, and gaps in social safety nets [2]. Together, these factors underscore food insecurity as a major public health issue with far-reaching implications for population health, health equity, and healthcare systems worldwide.

Although these determinants of food insecurity operate largely at the structural and societal level, their consequences are ultimately experienced at the level of individual health and physiology. Mounting evidence shows that food insecurity contributes to a wide spectrum of chronic diseases, poor mental health, and premature mortality, making it both a nutritional and a medical crisis [2]. Thus, while food insecurity is often framed as a socioeconomic issue, understanding it as a true biological stressor offers crucial insight into its health implications.

Despite growing recognition of food insecurity's role as a health determinant, the biological pathways mediating its effects remain fragmented across disciplines. The existing literature tends to be siloed within specific research domains, each emphasizing different aspects of the problem. For example, cardiologists often link food insecurity to hypertension, atherosclerosis, and cardiovascular mortality through pathways of chronic stress and inflammation [3], while endocrinologists and diabetes researchers emphasize insulin resistance, glycemic variability, and cycles of metabolic dysregulation [4]. Likewise, psychologists examine the emotional and behavioral consequences of food insecurity—such as anxiety, depression, and disordered eating—that further reinforce physiological stress responses [5]. Each of these perspectives offers valuable insights, yet the lack of integration across disciplines has limited our understanding of how these mechanisms converge at the biological level. As a result, the literature remains rich in descriptive associations but limited in mechanistic synthesis.

The consequences of this fragmentation are not merely academic. Without a cohesive biological framework, interventions may more often target isolated nutritional issues, such as improving diet quality, without addressing the physiological stress pathways that sustain health disparities. For example, a cardiology intervention might emphasize sodium reduction, while a nutrition policy may focus on caloric adequacy, and a behavioral program might aim to reduce depressive symptoms. Yet all these outcomes may share a common upstream driver: the chronic biological stress imposed by food insecurity. In this way, a patient may experience greater health benefits not by independently controlling sodium intake or taking medication to alleviate mental health burdens, but rather by addressing an underlying cause of food insecurity and its sequiturs. Thus, integrating these perspectives can clarify the causal pathways linking social deprivation to disease and improve the design of interventions that simultaneously address biological, behavioral, and social determinants of health.

**Fig. 1:**
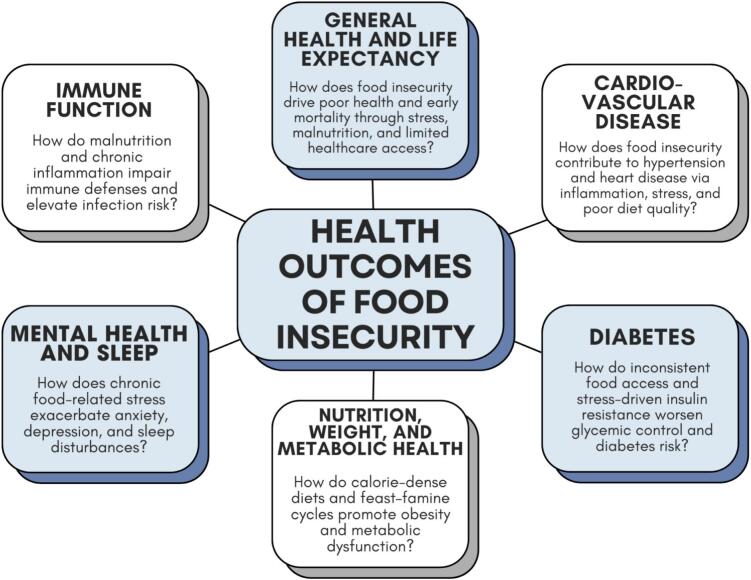
Integrative approach to synthesize the physiologic and psychosocial dysfunctions explored in this narrative review. This figure outlines six major domains through which food insecurity disrupts human health: general health and life expectancy, cardiovascular disease, diabetes, nutrition and metabolic function, mental health and sleep, and immune function. Each domain represents a distinct yet interconnected system illustrating how chronic stress, poor diet quality, and nutritional instability translate into multisystem biological dysfunction. Together, these areas form the framework for examining food insecurity as a complex, biologically embedded condition in this review.

To bridge this gap, an interdisciplinary synthesis is essential—one that combines the empirical breadth of epidemiologic research with the mechanistic depth of physiology and molecular biology. This narrative review was designed to do precisely that. By synthesizing evidence from nutrition, endocrinology, cardiology, immunology, and psychology, we aim to construct a framework that explains how food insecurity—a macroscopic socioeconomic phenomenon—influences health at the molecular, organ-system, and population levels. Specifically, this review examines six domains of health outcomes consistently linked to food insecurity: [1] general health and life expectancy, [2] cardiovascular disease, [3] diabetes, [4] nutrition, weight, and metabolic health, [5] mental health and sleep, and [6] immune function (Fig. 1). For each domain, we integrate epidemiologic findings with mechanistic explanations drawn from the scientific literature to illustrate the converging pathways through which food insecurity contributes to disease risk. We then describe key policy initiatives aimed at advancing health equity by breaking the biological link between food insecurity and chronic disease.

This narrative review fills a critical gap in the literature by framing food insecurity as a biologically embedded condition and synthesizing evidence across disciplines. Rather than describing health associations within isolated fields, it emphasizes shared physiological mechanisms like chronic nutritional stress, inflammation, and hormonal dysregulation that mediate these health effects across organ systems. Moreover, by identifying the shared biological mechanisms that underpin diverse health outcomes, we lay the foundation for more holistic policy and clinical interventions that treat food access as a determinant of physiological stability rather than a matter of caloric sufficiency alone. In doing so, this review contributes to a growing movement in medicine and public health that views food as medicine, recognizing that addressing food insecurity is not simply an act of social policy but a form of disease prevention at the most fundamental biological level.

This narrative review aligns with several World Health Organization (WHO) priorities and the United Nations Sustainable Development Goals (SDGs) that guide global public health efforts. Most directly, it supports SDG 2 (Zero Hunger) and SDG 10 (Reducing Inequalities) by emphasizing food insecurity as a critical determinant of nutrition and long-term health. It also aligns with SDG 3 (Good Health and Well-Being) by synthesizing evidence linking food insecurity to major noncommunicable diseases, including cardiovascular disease, diabetes, obesity, and mental health disorders [6]. By framing food insecurity as a biologically embedded stressor, this work reinforces global objectives aimed at improving nutrition security, reducing preventable disease burden, and advancing health equity.

## 2 Methods

This review was guided by two research questions designed to examine the multiple dimensions of the physiologic mechanisms through which food insecurity influences health outcomes:

a. What does the current literature state about the connection between food insecurity and health effects, including general health and life expectancy, cardiovascular disease, diabetes, obesity, mental health and sleep, and immune function?
b. What are the underlying biological mechanisms that mediate any observed connections between food insecurity and health outcomes?

Literature search: We adopted a comprehensive, narrative approach rather than a formal systematic review in order to synthesize evidence across disciplines of nutrition, physiology, endocrinology, psychology, and sociology. This approach was selected to allow integration of heterogeneous study designs and mechanistic data that may not be readily captured within a narrowly defined systematic framework. Additionally, because the objective of this review was conceptual and mechanistic synthesis rather than quantitative effect estimation, a narrative design was better suited to identifying shared biological pathways across diverse health outcomes. To capture the breadth of literature, a literature search was conducted through PubMed, Web of Science, and Scopus on August 15th, 2025, focusing on studies published worldwide between 1995 and 2025. See Table 1 for the full list of search terms used. Full database-specific search strategies, including all Boolean operators and filters, are provided in Supplementary Table S1.

**Table 1:** Database-specific search strategies used to identify studies examining associations between food insecurity and cardiometabolic, mental health, and sleep outcomes.

Database	Search String	Number of Studies
PubMed	(“food insecurity”[Title/Abstract]) AND (“cardiovascular disease”[Title/Abstract] OR diabetes[Title/Abstract] OR obesity[Title/Abstract] OR “mental health”[Title/Abstract] OR “sleep”[Title/Abstract]) NOT (agriculture[Title/Abstract] OR agricultural[Title/Abstract] OR crop*[Title/Abstract] OR soil[Title/Abstract] OR livestock[Title/Abstract] OR animal*[Title/Abstract] OR fisheries[Title/Abstract] OR farming[Title/Abstract])	2834
Web of Science	(“food insecurity”[Title/Abstract] OR “food insecure”[Title/Abstract]) AND ( “cardiovascular disease”[Title/Abstract] OR diabetes[Title/Abstract] OR obesity[Title/Abstract] OR “mental health”[Title/Abstract] OR sleep[Title/Abstract] ) NOT ( agriculture[Title/Abstract] OR agricultural[Title/Abstract] OR crop*[Title/Abstract] OR soil[Title/Abstract] OR livestock[Title/Abstract] OR animal*[Title/Abstract] OR fisheries[Title/Abstract] OR farming[Title/Abstract] )	9
Scopus	TITLE-ABS-KEY (“food insecurity”) AND (TITLE-ABS-KEY (“food insecurity” W/5 diabetes) OR TITLE-ABS-KEY (“food insecurity” W/5 “cardiovascular disease”) OR TITLE-ABS-KEY (“food insecurity” W/5 obesity) OR TITLE-ABS-KEY (“food insecurity” W/5 depression) OR TITLE-ABS-KEY (“food insecurity” W/5 anxiety)) AND PUBYEAR >1997 AND PUBYEAR <2026 AND (LIMIT-TO (DOCTYPE, “ar”) OR LIMIT-TO (DOCTYPE, “re”))	983

Study Selection and Deduplication: Records identified through database searches were compiled and screened for relevance. Duplicate records were removed where identified. Titles and abstracts were reviewed, followed by full-text assessment of selected articles based on their relevance to the research questions and biological focus of the review. Given the narrative design, study selection emphasized conceptual relevance rather than exhaustive inclusion.

Inclusion and Exclusion Criteria: We included studies that (a) examined associations between food insecurity and any of the six specified health outcomes, (b) described biological or physiological mechanisms linking food insecurity to these outcomes, and (c) were published in peer-reviewed journals or authoritative reports. We excluded articles that were not peer-reviewed; editorials, pre-prints, news articles; as well as sources that focused solely on food insecurity without addressing health outcomes or vice versa. Studies solely focusing on agriculture, crop production, livestock, fisheries, and soil were excluded in order to maintain the review's focus on food insecurity as a biologically embedded determinant of human disease rather than on food production systems or agricultural economics. Studies were limited to English-language publications to ensure authors could accurately interpret complex ideas. Eligible sources included original empirical studies (such as observational, experimental, qualitative, and mixed-methods research) as well as review articles linking food insecurity to health outcomes. Review articles were included to inform conceptual and mechanistic synthesis, but primary empirical studies were preferentially cited for specific epidemiologic and biological findings, and review conclusions were not treated as independent primary data. This ensured the review remained focused on the connection between food insecurity and its health consequences. Grey literature was not systematically included in this review, as the primary objective was to synthesize peer-reviewed evidence describing biological and physiological mechanisms linking food insecurity to health outcomes.

**Fig. 2:**
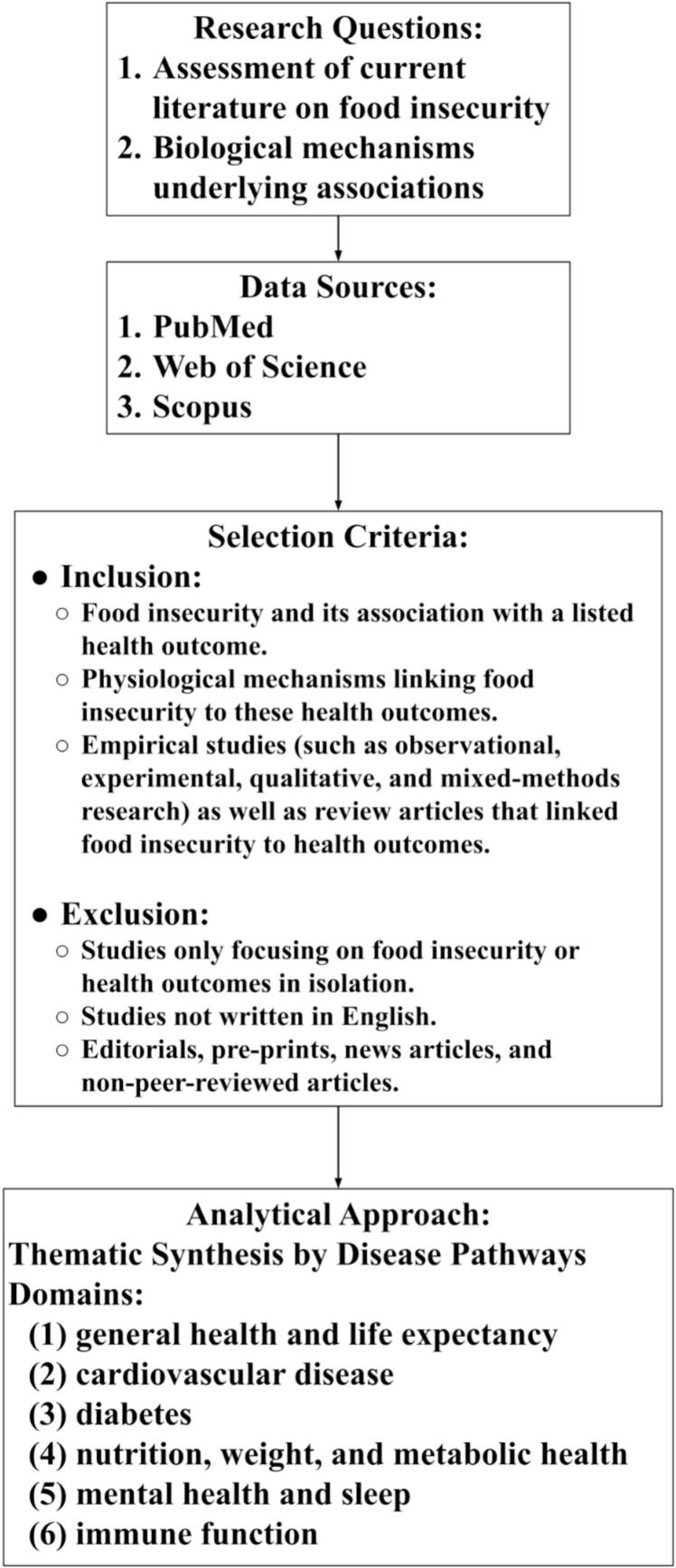
Conceptual framework of the narrative review. The diagram illustrates the progression from research questions through literature search, application of inclusion and exclusion criteria, determination of study scope, and thematic synthesis. This framework demonstrates how diverse sources were identified, screened, and organized into six domains (general health and life expectancy; cardiovascular disease and diabetes; nutrition, weight, and metabolic health; mental health and sleep; and immune function), providing a structured yet flexible approach aligned with the principles of a narrative review.

Analytical Approach: Findings were synthesized thematically across six predefined health domains, allowing for structured integration of evidence while maintaining the conceptual and mechanistic focus of this narrative review. Six health outcomes outlined in the research questions were: [1] general health and life expectancy, [2] cardiovascular disease, [3] diabetes, [4] nutrition, weight, and metabolic health, [5] mental health and sleep, and [6] immune function. Within each domain, we first describe the current literature describing food insecurity's connection to the health effect before outlining potential causal biological pathways underpinning the association. These outcomes were selected for three primary reasons: they represent high-burden, high-prevalence conditions, are supported by a comparatively mature mechanistic evidence base linking food insecurity to biological dysfunction, and collectively span the life course, affecting individuals from early childhood through older adulthood. Other conditions, such as chronic kidney disease, maternal-fetal outcomes, and cancer, were not included in this review because they either affect more specific subpopulations or currently have a less well-developed mechanistic literature directly connecting them to food insecurity. A formal, tool-based assessment of individual study quality was not performed, in line with the narrative review design and SANRA guidelines. The process is summarized in Fig. 2, which illustrates the integration of research questions, data sources, and thematic categories within the review framework. While this review follows a narrative design and does not aim for exhaustive systematic inclusion, a simplified flow diagram (Fig. 3) is provided to enhance transparency in the study identification and selection process. Given the narrative design of this review and the heterogeneity of included studies, a formal study-level data extraction table was not developed. Instead, studies were synthesized thematically to emphasize conceptual and mechanistic insights across health domains (as described above). Where relevant, distinctions between primary studies and review articles are noted within the text to avoid duplication of evidence. As this study was conducted as a narrative review, prospective protocol registration was not applicable. (See Fig. 3.)

**Fig. 3:**
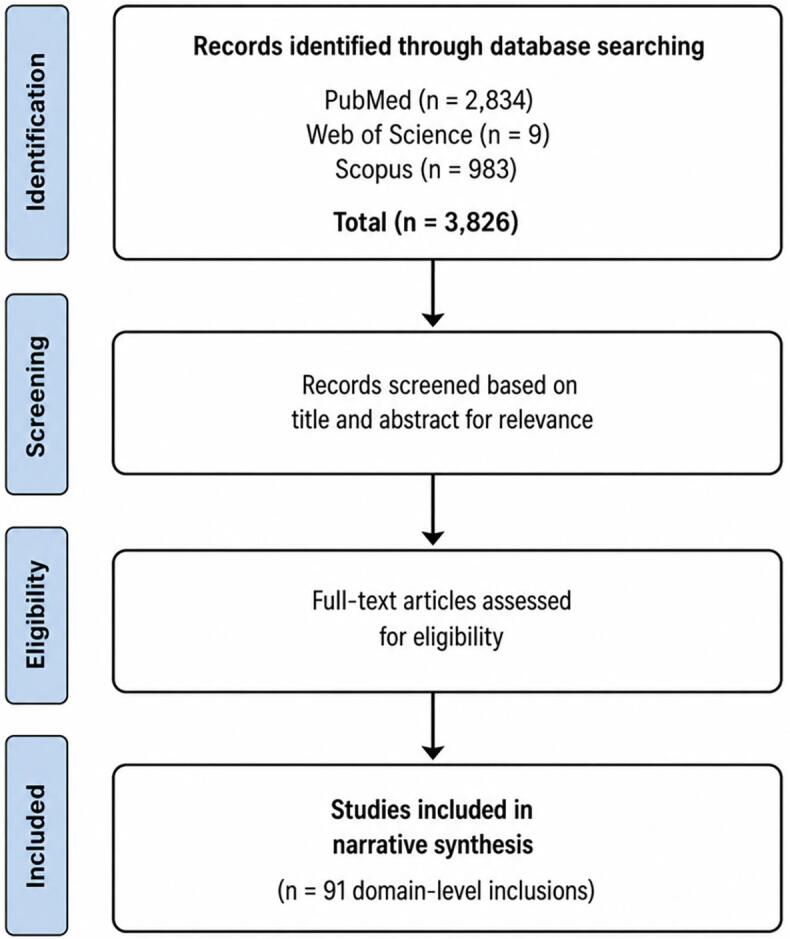
Flow diagram of study identification and selection. Records were identified through database searches (PubMed, Web of Science, and Scopus; *n* = 3826). Studies were screened based on title, abstract, and full-text relevance to the research questions. Eligible studies were included in the narrative synthesis across six health domains (*n* = 91 domain-level inclusions). This diagram is intended to enhance transparency and does not represent a formal systematic review workflow.

**Fig. 3:**
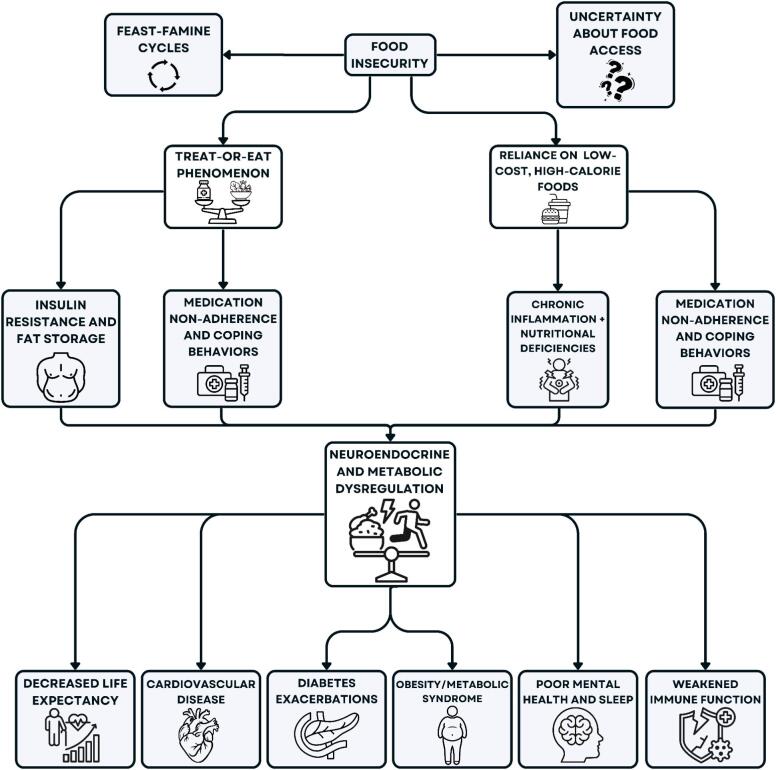
Mechanistic summary linking food insecurity to biological dysfunction and health outcomes. This figure depicts how food insecurity triggers behavioral and physiological disruptions, such as stress, inflammation, and metabolic dysregulation, that lead to multisystem health consequences across the six domains discussed in this review.

## 3 Results and discussion

### 3.1 Description of included studies

The final synthesis included 91 domain-level study inclusions across six predefined health categories. Specifically, 10 studies informed the section on general health and life expectancy, 17 addressed cardiovascular disease, 26 focused on diabetes, 10 examined nutrition, weight, and metabolic health, 18 addressed mental health and sleep, and 11 examined immune function. Because this review adopted a narrative framework, additional studies cited in the introduction as well as the policy discussion were deliberately sought out to provide contextual and mechanistic background rather than formally screened within domain-specific categories. Some studies were referenced across multiple sections when they addressed shared biological mechanisms or overlapping outcomes relevant to more than one health domain.

The majority of included studies used cross-sectional surveys, which provide thorough insights into food insecurity's co-existence with numerous health indicators among diverse populations (ex: [7], [8], [9], [10]). A smaller number of studies employed mixed methods, combining surveys with laboratory studies (ex: [11], [12]). Other studies used systematic review methods or meta-analyses to synthesize studies, providing context and mechanistic understanding (ex: [13], [14], [15]). Notably, considerable heterogeneity exists across included studies with regards to population characteristics (e.g., children [16] vs. adults [17]), measurement tools (e.g., using the Food and Physical Activity Questionnaire [18] vs. the Healthy Eating Index-2015 [19], and data sources used (ex: the National Health and Nutrition Examination Survey (NHANES) database [20] vs. the Medical Expenditure Panel Survey database [21]).

## 4 General health and life expectancy

### 4.1 Literature review

Food insecurity is consistently associated with poorer self-rated health and accelerated health decline across the lifespan [22]. Population-based studies show that food-insecure children face nearly twice the risk of poor health and higher rates of acute and chronic illness, while food-insecure older adults experience faster functional decline and greater difficulty with activities of daily living [22]. Beyond morbidity, longitudinal analyses demonstrate that food insecurity is associated with substantially increased all-cause mortality. Nationally representative cohort studies report 40–80% higher risks of premature death among adults with marginal or very low food security compared to food-secure adults, even after adjustment for socioeconomic and health factors [10], [23]. Collectively, this evidence indicates that inadequate food access is linked to degraded overall health and reduced survival.

### 4.2 Biological mechanisms

Several interrelated biological and psychosocial pathways plausibly explain these associations. Chronic nutritional insecurity functions as a persistent psychosocial stressor, activating the hypothalamic–pituitary–adrenal axis and sympathetic nervous system [22], [24]. Repeated activation of these systems promotes cortisol elevation, systemic inflammation, and metabolic dysregulation, which are hallmarks of increased allostatic load (i.e., cumulative physiological stress burden) [22], [24]. Empirical studies confirm that food-insecure individuals exhibit higher allostatic load scores, reflecting cumulative physiological strain across cardiovascular, metabolic, and immune systems, which is strongly associated with increased morbidity and mortality [24], [25]. Importantly, participation in nutrition assistance programs has been shown to attenuate this biological stress burden, suggesting that alleviating food insecurity may directly improve physiological regulation [25].

Nutritional inadequacy further contributes to poor general health. Food-insecure households often rely on calorie-dense, nutrient-poor foods and experience meal skipping, resulting in insufficient intake of protein, vitamins, and minerals [26], [27]. These deficiencies are associated with anemia, impaired immune function, and worsening of chronic disease states [26], [27]. In parallel, food insecurity forces difficult trade-offs between food, medications, and healthcare—a phenomenon commonly described as “treat or eat.” Food-insecure adults are significantly more likely to underuse prescribed medications and delay medical care due to cost, undermining disease management and increasing the risk of complications and hospitalization [27], [28], [29]. However, it is important to interpret this phenomenon within its broader societal context, as cost-related medication underuse is strongly influenced by the affordability of medications themselves. In countries with low out-of-pocket prescription costs, such as the Netherlands and the United Kingdom, cost-related medication underuse is reported far less frequently [30].

Together, chronic stress exposure, nutritional deficiency, and constrained healthcare access create a reinforcing cascade of biological and behavioral risk that may contribute to accelerated health decline and increased risk of premature death among food-insecure populations.

## 5 Cardiovascular disease

### 5.1 Literature review

Food insecurity is consistently associated with elevated cardiovascular risk, including higher rates of hypertension, heart disease, and cardiovascular mortality [3], [23], [31]. Population-based and prospective studies demonstrate that food-insecure adults have greater prevalence of multiple cardiovascular conditions and a substantially higher risk of cardiovascular death compared to food-secure individuals, even after adjustment for socioeconomic and lifestyle factors [3], [23], [31].

Beyond mortality, food insecurity is linked to poorer control of cardiovascular risk factors, including hypertension and hyperlipidemia, as well as reduced adherence to antihypertensive medications and inconsistent access to care. These constraints complicate chronic disease management and contribute to higher hospitalization rates for cardiovascular events [3], [11], [28], [32], [33], [34].

Food insecurity is also associated with behaviors and dietary patterns that further increase risk for hypertension and atherosclerosis, including higher smoking prevalence, lower physical activity, and reliance on low-cost, sodium- and fat-dense foods [3], [32], [35], [36]. Taken together, the evidence indicates that food insecurity functions as a robust marker of elevated cardiovascular risk through intertwined biological, behavioral, and healthcare access pathways [3], [32], [35], [36].

### 5.2 Biological mechanisms

Several interrelated biological and behavioral pathways help explain how food insecurity contributes to poor cardiovascular health [37]. Food-insecure households often rely on low-cost, calorie-dense foods high in refined carbohydrates, saturated fats, and sodium, and low in fruits, vegetables, and whole grains [3], [37], [38]. This dietary pattern is thought to promote hypertension, dyslipidemia, obesity, and insulin resistance, which serve as key risk factors for cardiovascular disease by impairing vascular function through micronutrient deficiencies and endothelial dysfunction [39], [40], [41], [42]. Chronic stress and elevated allostatic load, discussed in detail in the “General Health and Life Expectancy” section, further contribute by increasing blood pressure, heart rate, systemic inflammation, and levels of circulating stress hormones like cortisol and adrenaline [25], [43]. Collectively, these effects may contribute to atherogenesis and are associated with increased risk of coronary artery disease, myocardial infarction, and heart failure [44].

Behavioral adaptations also play a role. In addition to utilizing coping behaviors, food-insecure individuals may delay care or underuse medications for hypertension or heart failure due to competing financial demands, which is associated with poorer disease control and higher hospitalization rates [28], [33], [36], [45], [46]. These converging pathways demonstrate how food insecurity translates into sustained cardiovascular vulnerability beyond traditional risk factors.

## 6 Diabetes

### 6.1 Literature review

Substantial evidence links food insecurity to both the development and progression of type 2 diabetes [21], [47]. Cross-sectional and longitudinal studies consistently identify food insecurity as a risk factor for diabetes, with a large meta-analysis reporting 27% higher odds of type 2 diabetes among adults in food-insecure households [13]. In addition, nearly one-third of Medicaid enrollees with diabetes experience food insecurity, emphasizing the prevalence of co-occurring socioeconomic effects with metabolic disease [21].

Beyond disease onset, food insecurity is strongly associated with poorer glycemic control and diabetes management [48]. Food-insecure individuals with diabetes exhibit higher HbA1c levels and greater glycemic variability, increasing the risk of acute complications such as hyperglycemic crises and hypoglycemia-related hospitalizations [48], [49], [50], [51]. This pattern reflects a well-described “diabetes–food insecurity cycle,” in which inconsistent access to food disrupts glucose regulation and self-management behaviors [52], [53]. For example, low-income patients exhibited increased hypoglycemia-related hospital admissions near the end of the month—when food budgets were likely depleted—underscoring the direct physiological effects of food scarcity on diabetes outcomes [53].

Additionally, research has found that food insecurity is correlated with key risk factors for diabetes, such as obesity and metabolic syndrome [54], [55]. Moreover, food-insecure individuals with diabetes often face the “treat or eat” phenomenon previously discussed: studies report that food-insecure adults with chronic illness are more likely to underuse medications (like insulin or oral hypoglycemics) to save money for food, worsening glycemic control and increasing complication risk [28], [56], [57], [58]. Thus, the literature suggests that food insecurity is associated with higher risk of developing diabetes and, among those with diabetes, it predicts greater difficulty in managing the disease and a higher incidence of adverse outcomes.

### 6.2 Biological mechanisms

Food insecurity contributes to the development and progression of diabetes through intertwined nutritional, biological, and behavioral pathways. Limited resources often lead to reliance on inexpensive, energy-dense foods and irregular eating patterns, promoting weight gain, central adiposity, insulin resistance, and repeated glycemic excursions that strain pancreatic β-cell function [55], [59], [60], [61], [62], [63]. Poor diet quality and unhealthy cooking practices further exacerbate metabolic dysregulation and pancreatic strain via blood glucose spikes, fostering conditions conducive to type 2 diabetes [19], [64], [65], [66], [67].

Chronic psychosocial stress represents a complementary mechanism. Persistent uncertainty about food access activates stress-response systems, increasing cortisol and sympathetic activity, which elevate blood glucose through enhanced gluconeogenesis, lipolysis, and insulin resistance [68], [69]. Thus, through overlapping nutritional, metabolic, and behavioral pathways, food insecurity worsens diabetes risk and glycemic regulation.

## 7 Nutrition, weight, and metabolic health

### 7.1 Literature review

Obesity remains highly prevalent among U.S. adults, with recent estimates indicating a prevalence of 40.3% [70]. The co-occurrence of food insecurity and obesity reflects a well-documented public health paradox, as food insecurity is consistently associated with increased risk of overweight and obesity, particularly among women [40], [55], [71], [72], [73]. Population-based studies report that as food insecurity severity increased, the odds of obesity rose significantly among women, with food-insecure adults having 32% increased odds of being obese compared to their food-secure counterparts [74]. Moreover, some studies find higher obesity rates in food-insecure youth once they reach adolescence, signifying the lasting effects of inadequate access to nutritious food [55], [75]. Food-insecure individuals also report lower engagement in health-promoting food behaviors, including less frequent home cooking, reduced use of nutrition labels, and lower self-efficacy for healthy eating [18]. In combination, persistent dietary inadequacy and constrained food-related behaviors help explain the elevated obesity risk observed in food-insecure populations.

### 7.2 Biological mechanisms

Food insecurity contributes to obesity and metabolic dysfunction through interconnected dietary, behavioral, and hormonal pathways. First, cost constraints often drive reliance on energy-dense, nutrient-poor foods high in refined carbohydrates, added sugars, and unhealthy fats—a pattern consistent with excess caloric intake despite inadequate micronutrient intake and periods of hunger [38], [40], [76], [77].

Irregular eating patterns further exacerbate metabolic risk. The commonly described “feast–famine” cycle, defined by periods of overeating following food scarcity, is thought to promote fat storage, insulin resistance, and abdominal adiposity, reflecting metabolic adaptations to unreliable food availability [61], [78]. In essence, irregular food intake patterns and reliance on cheap calories in food insecurity set the stage for metabolic “thriftiness” that manifests as obesity [78].

Chronic psychosocial stress also plays a central role. Elevated cortisol associated with food insecurity increases visceral fat deposition and preference for high-calorie “comfort foods,” reinforcing weight gain [76], [78]. Likewise, children in food-insecure environments might develop a preference for energy-dense foods or a tendency to overeat when food is available, as an adaptive response, explaining why early-life exposure to food insecurity may increase susceptibility to obesity later in life [55], [75].

Together, erratic food access, poor diet quality, stress-induced hormonal dysregulation, and nutrient insufficiency contribute to a pattern of being “overfed but undernourished,” explaining the paradoxical association between food insecurity and obesity observed across populations.

## 8 Mental health and sleep

### 8.1 Literature review

Food insecurity is widely recognized as a chronic psychosocial stressor that undermines emotional well-being and is strongly associated with depression, anxiety, psychological distress, and suicidality across the life course [14], [79], [80], [81]. Meta-analytic and large population-based studies consistently report higher odds of depression and stress among food-insecure individuals, with particularly elevated risk observed among young adults and economically vulnerable populations [14], [79], [80], [81]. These associations extend beyond individuals to families, as parents in food-insecure households experience higher rates of depression and anxiety, while children exposed to food insecurity show increased emotional and behavioral problems [22], [82].

Food insecurity is also consistently linked to poor sleep outcomes, including shorter sleep duration, insomnia, sleep fragmentation, and dysregulated sleep patterns, suggesting disruption of circadian regulation [7], [9], [80]. Mental health and sleep disturbances are closely intertwined, forming a reinforcing cycle in which food insecurity-related stress worsens sleep, and sleep disruption exacerbates emotional distress and anxiety about food access [14], [80]. Sex- and gender-based differences in stress physiology and sleep regulation may also influence these associations, although these factors are not consistently examined in the literature [81]. This evidence highlights food insecurity as a robust social determinant of both mental health and sleep across diverse populations.

### 8.2 Biological mechanisms

The association between food insecurity, mental health, and sleep disturbances is driven primarily by chronic psychosocial stress [83], [84]. Uncertainty about food access activates the hypothalamic-pituitary-adrenal axis, elevating cortisol, disrupting neurotransmitter systems involved in mood regulation, and impairing circadian rhythms [84]. This dysregulation increases risk for depression, anxiety, and insomnia [85]. Neuroimaging studies further link food insecurity-related stress to heightened amygdala activity associated with fear, anxiety, and poor sleep quality [86], [87].

Nutritional deficiencies compound these effects. Food insecurity is associated with lower intake of nutrients essential for brain and sleep function, including omega-3 fatty acids, B vitamins, iron, and magnesium [88], [89], [90]. These deficiencies contribute to depression, cognitive impairment, fatigue, restless sleep, and insomnia [88], [89], [90].

Psychosocial and behavioral feedback loops further reinforce these outcomes. Parents experiencing food insecurity report higher stress and depression, while children show behavioral and emotional problems [22], [91]. In turn, mental health issues like depression can cause changes in appetite and energy that feed back into the cycle of food insecurity. Sleep loss also impairs neurotransmitter recovery, increasing mood disorders and suicidality [92], [93].

Such interconnected pathways reveal how food insecurity drives neuroendocrine, nutritional, and sleep-related disruptions that elevate mental illness risk, emphasizing the need for integrated food and mental health interventions.

## 9 Immune function

### 9.1 Literature review

Food insecurity is consistently associated with heightened inflammation and impaired immune function. Population-based studies link food insecurity to elevated inflammatory biomarkers, including C-reactive protein (CRP), proinflammatory cytokines, and increased white blood cell counts, indicating chronic low-grade inflammation across diverse populations [94], [95]. Chronic inflammation associated with food insecurity and psychosocial stress is a well-established contributor to the development of multiple chronic diseases, including cardiovascular disease, diabetes, cancer, chronic kidney disease, and neurodegenerative disorders [96].

In low- and middle-income countries, food insecurity frequently manifests as malnutrition, which directly compromises immune competence and increases susceptibility to infection [97], [98]. Undernourished children experience impaired innate and adaptive immune responses, which are associated with higher rates of infectious disease and mortality, even in cases of mild undernutrition [97], [98]. Studies from low- and middle-income countries demonstrate a dose-response relationship between food insecurity severity and infection risk, underscoring that inadequate or insecure nutrition undermines immune defenses across settings and populations [99], [100]. These global patterns underscore that inadequate or insecure nutrition, in any population, may undermine normal immune defenses and increase susceptibility to disease.

### 9.2 Biological mechanisms

Food insecurity is associated with impaired immune function through interconnected pathways of nutritional deficiency and gut dysregulation. Adequate macro- and micronutrient intake is essential for normal immune cell development and function, and food insecurity is associated with insufficient protein, calorie, and micronutrient intake that weakens host defenses and increases susceptibility to infection [101], [102]. For example, nutrient deficiencies have been shown to impair lymphocyte production, antibody responses, and inflammatory regulation, contributing to immune dysfunction [101], [102]. Insufficient nutrition may also lead to alterations in the architecture of the gut mucosa, such as flattened microvilli, reduced lymphocyte counts in Peyer's patches, and reduced IgA production [102].

Alterations in the gut microbiome further link food insecurity to immune impairment [97], [103]. Diets low in diversity and fiber promote microbial dysbiosis and compromise gut barrier integrity, allowing microbial products to translocate into the bloodstream and trigger chronic immune activation [97], [103]. This gut-mediated inflammation reinforces a cycle of infection, impaired nutrient absorption, and immune dysregulation. This microbial translocation continuously triggers the immune system, resulting in systemic inflammation and immune activation even in the absence of overt infection [97], [104]. Gut barrier breakdown thus contributes to malnutrition's vicious cycle: infections become more frequent, nutrients are less absorbed, and inflammatory signals increase [97]. Thus, nutrient insufficiency and gut-derived inflammation may converge to help explain how food insecurity increases vulnerability to infection and inflammatory disease.

## 10 Policy interventions: Examples from the United States

Policy interventions are urgently needed to counteract the physiological consequences of food insecurity, which disrupt metabolic stability and elevate chronic disease risk [41]. Mitigating these health effects at the microscopic level of human metabolism demands coordinated, macroscopic action—uniting healthcare systems, public health agencies, and community organizations to ensure consistent access to nutritious food. Addressing food insecurity thus requires comprehensive policy collaboration that bridges biological science and social infrastructure to promote lasting health equity.

The Supplemental Nutrition Assistance Program (SNAP), the nation's largest food assistance program, offers monthly benefits to help low-income households buy food [105]. Participation in SNAP is associated with improved food security and reduced chronic activation of stress-response pathways, including elevated cortisol, impaired glucose regulation, and increased allostatic load [24], [25]. Thus, SNAP participation supports SDG 2 (Zero Hunger) by improving food access and SDG 3 (Good Health and Well-Being)) by reducing biologically mediated disease risk. The 2021 update to the USDA's Thrifty Food Plan raised SNAP benefits by 21%, helping offset rising costs and limit health disparities, though benefit adequacy remains a concern for maintaining balanced diets [106], [107].

While SNAP participation is linked to lower odds of experiencing low food security and improved self-rated health, evidence shows that dietary quality among participants often remains suboptimal, with higher consumption of ultra-processed foods and sugar-sweetened beverages compared to income-eligible nonparticipants [108], [109], [110]. This partially stems from limited benefits and few incentives for healthy food choices within the program. The American Heart Association (AHA) recommends policy changes to incentivize nutritious food purchases (e.g., subsidizing fruits and vegetables) and disincentivize unhealthy options to strengthen SNAP's nutritional impact [111], [112].

Participation in the Special Supplemental Nutrition Program for Women, Infants, and Children (WIC) is associated with improvements in birth outcomes, reduction in infant mortality, and enhancements in neurodevelopment by providing access to healthy foods such as fruits, vegetables, whole grains, and low-fat dairy [113], [114], [115], [116]. WIC participation during pregnancy is linked to reduced risks of preterm birth, low birthweight, and infant mortality, with benefits seen across racial and ethnic groups [113], [115]. These outcomes are driven by biological mechanisms: enhanced maternal intake of protein, iron, and vitamin D promotes fetal growth and immune-endocrine development, lowering the likelihood of hypertension, preeclampsia, and fetal neurodevelopmental complications [117], [118]. Early-life nutrition through WIC contributes to shaping immune and metabolic programming, mitigating long-term vulnerability to metabolic disease [117], [118]. Despite these benefits, many eligible families remain unenrolled due to administrative barriers. Simplifying digital enrollment and expanding culturally competent outreach are essential to increase WIC participation and ensure these biologically protective effects reach those most at risk [119].

School nutrition programs, including the National School Lunch and School Breakfast Programs, are a frontline defense against the physiological consequences related to food insecurity. They provide millions of children with reliable access to nutrient-dense meals, allowing for improvements in metabolic health and reduction in obesity risk, particularly among low-income students [120], [121]. Nutritionally adequate school meals help stabilize glucose levels, support regular sleep, and reduce behavioral dysregulation, which directly affect learning, mental health, and overall development [122]. Studies show that universal meal policies are associated with lower obesity prevalence and improved attendance, especially for children at risk of food insecurity [123], [124]. For example, during the COVID-19 pandemic, universal free meal policies greatly reduced child food insecurity, boosted participation, and lessened stigma and financial stress, improving psychosocial and academic outcomes [125], [126]. By adopting permanent universal meal programs, several states are making a transformative investment in pediatric health equity in alignment with SDG 10 (Reducing Inequalities). Further, they are advancing the AHA's call to expand access, strengthen nutrition standards, and reduce disparities in nutrition security and chronic disease risk [112].

“Food is Medicine” (FIM) initiatives, such as medically tailored meals, groceries, and produce prescriptions, integrate nutrition interventions into healthcare to address diet-related disease and food insecurity [127], [128]. Prescribed by clinicians and guided by dietitians, these programs are increasingly reimbursed by Medicaid, Medicare Advantage, and commercial insurers [128], [129]. FIM interventions may contribute to improving key biological processes by enhancing diet quality, stabilizing glucose, and lowering allostatic load, reducing risks for obesity, diabetes, hypertension, and heart disease [128]. AHA reviews show these programs enhance food security, glycemic control, blood pressure, and overall health, with evidence of fewer hospitalizations. Produce prescription programs specifically increase fruit and vegetable intake among patients with type 2 diabetes and food insecurity [128], [129]. Federal policy and clinical guidelines support expanding FIM as an effective strategy to reduce inflammation, improve metabolic control, and promote health equity [128], [130].

Expanding nutrition policy interventions is critical to advancing health equity. SNAP, WIC, School Nutrition Programs, and Food Is Medicine initiatives indirectly target key physiological pathways associated with inadequate nutrition and chronic disease. Robust evidence supports continued investment and policy expansion of these programs as vital strategies to strengthen population health, prevent chronic disease, and reduce nutrition-related disparities. Implementing these interventions at scale is critical to build a more resilient, equitable health system that treats access to nutritious food as a fundamental determinant of well-being.

## 11 Conclusion

This review highlights food insecurity as a biologically embedded condition that affects health across multiple systems (see Table 2 for summary). Significantly, these findings reinforce the relevance of this work to global health priorities, particularly SDG 2 (Zero Hunger), SDG 3 (Good Health and Well-Being), and SDG 10 (Reduced Inequalities). Beyond social and economic hardship, food insecurity acts as a chronic physiological stressor—activating neuroendocrine, metabolic, and inflammatory pathways that are associated with increased risk of disease. The resulting allostatic load accelerates the development of cardiovascular disease, diabetes, obesity, poor mental health, and immune dysfunction. These findings underscore that food access is not merely a social issue but a determinant of biological stability and longevity (Fig. 3).

**Table 2:** Summary of studies examining food insecurity across major health domains.

Health Domain	Key Findings with Quantitative Insight	Biological Mechanisms Identified	Supporting Studies
General Health & Life Expectancy	Food insecurity is associated with poorer self-rated health and ∼ 40–80% higher risk of premature mortality among adults.	Chronic stress → elevated cortisol; increased allostatic load; nutritional deficiencies; delayed healthcare use.	[23], [25], [26], [28], [29]
Cardiovascular Disease	Higher prevalence of hypertension, coronary heart disease, and cardiovascular mortality; food insecurity is associated with elevated cardiovascular risk and poorer risk factor control.	Diet quality deficits; systemic inflammation and endothelial dysfunction; stress-mediated sympathetic activation; medication nonadherence.	[3], [23], [31], [33], [34]
Diabetes	Food insecurity is associated with ∼27% higher odds of type 2 diabetes and poorer glycemic control (e.g., higher HbA1c, increased hypoglycemia risk).	Insulin resistance; glycemic variability; stress-induced hyperglycemia; inconsistent medication use.	[13], [21], [49], [53], [55]
Nutrition, Weight & Metabolic Health	Food insecurity is associated with ∼32% higher odds of obesity, particularly among women, alongside poor diet quality and feast–famine eating cycles.	Metabolic adaptation to scarcity; cortisol-driven fat storage; nutrient insufficiencies.	[40], [73], [76], [77], [78]
Mental Health & Sleep	Food insecurity is associated with significantly higher odds of depression, anxiety, and sleep disturbances, with consistent findings across meta-analyses and population studies.	HPA-axis dysregulation; neurotransmitter disruption; micronutrient deficiencies; circadian misalignment.	[7], [80], [82], [87], [90]
Immune Function	Food insecurity is associated with elevated inflammatory markers (e.g., CRP, cytokines) and increased susceptibility to infection, particularly in undernourished population	Micronutrient deficiencies; microbiome dysbiosis; increased gut permeability; systemic inflammatory activation.	[100], [101], [102], [103], [104]

It is important to note that the transferability and generalizability of these findings warrant careful consideration, particularly for underserved populations who experience compounded structural vulnerabilities. While the biological mechanisms described in this review—chronic stress activation, inflammation, metabolic dysregulation, and immune impairment—are likely broadly applicable across contexts characterized by nutritional instability, the magnitude and manifestation of these effects may vary depending on healthcare access, social safety nets, cultural dietary patterns, and exposure to structural inequities. Populations facing intersecting stressors such as poverty, discrimination, housing instability, and limited preventive care may experience amplified physiological consequences. Therefore, although the mechanistic framework presented here is broadly relevant, its clinical and policy implications must be adapted to the social and economic realities of diverse and underserved communities to ensure equitable impact.

By integrating insights from nutrition science, endocrinology, cardiology, immunology, and behavioral health, this review demonstrates that food insecurity is not a single-issue problem but a multisystem disruption requiring coordinated solutions. Cross-disciplinary synthesis allows for a fuller appreciation of food insecurity's biological plausibility and underscores the need for interventions that operate simultaneously at biological, behavioral, and structural levels. Recognizing food as both medicine and infrastructure reframes food policy as a health intervention rather than a welfare measure.

Ultimately, the biology of scarcity calls for the biology of collaboration. Understanding food insecurity as a biologically embedded condition demands that researchers, clinicians, policymakers, and community leaders work in concert rather than in separate spheres. Physicians and biomedical scientists can illuminate the physiological mechanisms through which nutritional instability becomes disease; social scientists can contextualize those mechanisms within systems of inequity; and policymakers can translate this evidence into interventions that address both biology and circumstance. Such integration transforms fragmented observations into a cohesive framework capable of driving systemic change. By bridging the laboratory, the clinic, and the community, interdisciplinary collaboration can generate solutions that not only treat the symptoms of food insecurity but dismantle the structural conditions that sustain it. Addressing food insecurity through this collective lens is not only a moral imperative but a scientific one, essential for building a future where equitable access to nourishment is recognized as foundational to both human health and human dignity.

## 12 Strengths

This review's primary strength lies in its interdisciplinary synthesis of evidence spanning nutrition, physiology, endocrinology, cardiology, and psychology, providing a biologically grounded understanding of how food insecurity affects health. By integrating findings across six major health domains, the review highlights consistent physiological pathways—such as chronic stress, inflammation, and metabolic dysregulation—that link food insecurity to disease. The inclusion of studies utilizing large, nationally representative datasets like NHANES enhances the generalizability and robustness of the conclusions.

## 13 Limitations

However, several limitations should be acknowledged. As a narrative review, the synthesis may be influenced by selection bias and cannot establish causality. Many included studies rely on cross-sectional designs and self-reported measures of food insecurity and health outcomes, introducing potential recall or measurement error. The search strategy focused on the term “food insecurity,” which may have excluded studies using alternative terminology such as “food security” to describe similar exposure gradients, potentially limiting the breadth of captured literature. Considerable heterogeneity exists across studies in terms of population characteristics, measurement tools, and databases used, which may limit comparability. The restriction to English-language publications may have excluded relevant studies conducted in non-English-speaking settings, potentially limiting the global generalizability of findings. Likewise, the exclusion of grey literature may have limited inclusion of relevant policy and population-level insights not captured in peer-reviewed sources. Biological mechanisms discussed in the literature are often inferred rather than directly tested, reflecting a shortage of longitudinal and experimental research linking physiological pathways to food insecurity. Additionally, most evidence comes from U.S.-based populations, limiting generalizability to global or marginalized contexts. Differences in food systems, healthcare infrastructure, cultural dietary patterns, and social safety nets may influence both the experience of food insecurity and its biological consequences. As a result, the magnitude and manifestation of these physiological pathways may vary across settings, particularly in low- and middle-income countries. Food insecurity frequently overlaps with other social determinants, making it difficult to disentangle its independent biological effects. The chosen inclusion window (1995–2025) was selected to balance contemporary evidence with foundational studies; however, inclusion of older studies limited reliance exclusively on the most recent data. Finally, publication bias and limited mechanistic evaluation in policy studies may influence the conclusions drawn. Despite these limitations, this review provides a comprehensive and integrative foundation for understanding the biological underpinnings of food insecurity and guiding future interdisciplinary research. Additionally, as this review did not include a formal study-level extraction table, some study characteristics are described narratively rather than in a standardized tabular format.

## 14 Future directions

Future research should address key limitations in the current evidence base by prioritizing longitudinal and experimental study designs capable of clarifying causality between food insecurity and physiological dysfunction. Additionally, greater use of objective indicators of physiologic distress, including clinical biomarkers of inflammation, neuroendocrine stress, metabolic dysfunction, and immune regulation, could reduce reliance on self-reported measures and strengthen mechanistic conclusions. Expanding research beyond U.S.-based samples is also critical to improve generalizability and to better understand food insecurity in diverse cultural, economic, and policy contexts. Future studies may also examine how the effects of food insecurity vary across populations, including infants, pregnant and postpartum individuals, older adults, and other vulnerable subgroups, to better understand differential biological and clinical impacts across the life course. Finally, studies should evaluate whether food and policy interventions produce measurable biological changes, strengthening the translation of mechanistic insights into scalable, equity-driven solutions.

## CRediT authorship contribution statement

**Liahm Blank:** Writing – review & editing, Writing – original draft, Visualization, Validation, Supervision, Software, Resources, Project administration, Methodology, Investigation, Funding acquisition, Formal analysis, Data curation, Conceptualization. **Joshua Khorsandi:** Writing – review & editing, Writing – original draft, Validation, Methodology, Investigation, Formal analysis. **Nicole Kang:** Writing – review & editing, Writing – original draft, Methodology, Investigation. **Kaloyan Momchilov:** Writing – review & editing, Writing – original draft. **Itai Blank:** Writing – review & editing, Writing – original draft. **Kavita Batra:** Writing – review & editing, Validation, Supervision, Resources, Project administration, Methodology, Investigation, Formal analysis, Data curation, Conceptualization.

## Ethics approval and consent to participate

Not applicable.

## Funding

This review received no external funding.

## Declaration of competing interest

The authors declare no conflicts of interest.

## Data Availability

No new data were generated for this study. All data supporting the findings of this review, including search strategies, are included within the article and its supplementary materials.

## Appendix A Supplementary data

Table S1. Full database search strategies and applied filters used for literature retrieval across PubMed, Web of Science, and Scopus databases (1995–2025).
