# Supplementary material for: From pantry to physiology: A narrative review linking food insecurity to biological dysfunction
Source: Dialogues Health. 2026 May 12;8:100308. doi: 10.1016/j.dialog.2026.100308 (PMC13200126; doi:10.1016/j.dialog.2026.100308)
Supplement: Supplementary file 1 — Table S1. Full database search strategies and applied filters used for literature retrieval across PubMed, Web of Science, and Scopus databases (1995–2025). [file mmc1.docx]

| **Database** | **Full Search Strategy** | **Filters Applied** |
| --- | --- | --- |
| **PubMed** | ("food insecurity"[Title/Abstract]) AND ("cardiovascular disease"[Title/Abstract] OR diabetes[Title/Abstract] OR obesity[Title/Abstract] OR "mental health"[Title/Abstract] OR sleep[Title/Abstract]) NOT (agriculture[Title/Abstract] OR agricultural[Title/Abstract] OR crop*[Title/Abstract] OR soil[Title/Abstract] OR livestock[Title/Abstract] OR animal*[Title/Abstract] OR fisheries[Title/Abstract] OR farming[Title/Abstract]) | Language: English; Publication years: 1995–2025 |
| **Web of Science** | ("food insecurity"[Title/Abstract] OR "food insecure"[Title/Abstract]) AND ("cardiovascular disease"[Title/Abstract] OR diabetes[Title/Abstract] OR obesity[Title/Abstract] OR "mental health"[Title/Abstract] OR sleep[Title/Abstract]) NOT (agriculture[Title/Abstract] OR agricultural[Title/Abstract] OR crop*[Title/Abstract] OR soil[Title/Abstract] OR livestock[Title/Abstract] OR animal*[Title/Abstract] OR fisheries[Title/Abstract] OR farming[Title/Abstract]) | Language: English; Publication years: 1995–2025 |
| **Scopus** | TITLE-ABS-KEY ("food insecurity") AND (TITLE-ABS-KEY ("food insecurity" W/5 diabetes) OR TITLE-ABS-KEY ("food insecurity" W/5 "cardiovascular disease") OR TITLE-ABS-KEY ("food insecurity" W/5 obesity) OR TITLE-ABS-KEY ("food insecurity" W/5 depression) OR TITLE-ABS-KEY ("food insecurity" W/5 anxiety)) | Language: English; Publication years: 1995–2025 |

**Table S1: Full Database Search Strategies**
